# Efficacy of Ultra-Early (< 12 h), Early (12–24 h), and Late (>24–138.5 h) Surgery with Magnetic Resonance Imaging-Confirmed Decompression in American Spinal Injury Association Impairment Scale Grades A, B, and C Cervical Spinal Cord Injury

**DOI:** 10.1089/neu.2019.6606

**Published:** 2020-01-09

**Authors:** Bizhan Aarabi, Noori Akhtar-Danesh, Timothy Chryssikos, Kathirkamanathan Shanmuganathan, Gary T. Schwartzbauer, J. Marc Simard, Joshua Olexa, Charles A. Sansur, Kenneth M. Crandall, Harry Mushlin, Matthew J. Kole, Elizabeth J. Le, Aaron P. Wessell, Nathan Pratt, Gregory Cannarsa, Cara Lomangino, Maureen Scarboro, Carla Aresco, Jeffrey Oliver, Nicholas Caffes, Stephen Carbine, Kanami Mori

**Affiliations:** ^1^ Department of Neurosurgery, University of Maryland School of Medicine, Baltimore, Maryland.; ^2^ R. Adams Cowley Shock Trauma Center, University of Maryland School of Medicine, Baltimore, Maryland.; ^3^ School of Nursing and Department of Health Research Methods, Evidence, and Impact, McMaster University, Hamilton, Ontario, Canada.

**Keywords:** decompression, MRI, outcome, SCI, timing of surgery

## Abstract

In cervical traumatic spinal cord injury (TSCI), the therapeutic effect of timing of surgery on neurological recovery remains uncertain. Additionally, the relationship between extent of decompression, imaging biomarker evidence of injury severity, and outcome is incompletely understood. We investigated the effect of timing of decompression on long-term neurological outcome in patients with complete spinal cord decompression confirmed on postoperative magnetic resonance imaging (MRI). American Spinal Injury Association (ASIA) Impairment Scale (AIS) grade conversion was determined in 72 AIS grades A, B, and C patients 6 months after confirmed decompression. Thirty-two patients underwent decompressive surgery ultra-early (< 12 h), 25 underwent decompressive surgery early (12–24 h), and 15 underwent decompressive surgery late (> 24–138.5 h) after injury. Age, gender, injury mechanism, intramedullary lesion length (IMLL) on MRI, admission ASIA motor score, and surgical technique were not statistically different among groups. Motor complete patients (*p* = 0.009) and those with fracture dislocations (*p* = 0.01) tended to be operated on earlier. Improvement of one grade or more was present in 55.6% of AIS grade A, 60.9% of AIS grade B, and 86.4% of AIS grade C patients. Admission AIS motor score (*p* = 0.0004) and pre-operative IMLL (*p* = 0.00001) were the strongest predictors of neurological outcome. AIS grade improvement occurred in 65.6%, 60%, and 80% of patients who underwent decompression ultra-early, early, and late, respectively (*p* = 0.424). Multiple regression analysis revealed that IMLL was the only significant variable predictive of AIS grade conversion to a better grade (odds ratio, 0.908; confidence interval [CI], 0.862–0.957; *p* < 0.001). We conclude that in patients with post-operative MRI confirmation of complete decompression following cervical TSCI, pre-operative IMLL, not the timing of surgery, determines long-term neurological outcome.

## Introduction

The pathophysiology of cervical traumatic spinal cord injury (TSCI) is complex, and there remains no effective treatment for this high-impact disorder.^1–6^ In cervical TSCI, there is disruption of the anatomic integrity of the vertebral column followed by endothelial, neuronal, and axonal damage within fractions of a second of the injury. Continued compression of the spinal cord during the ensuing hours culminates in ischemia, swelling, and hemorrhagic progression of a compressive contusion.^7–9^ In this scenario, a deleterious cycle of events ensues, in which molecular cascades instigate the upregulation of cationic channels that promote secondary injury and edema, which is visible on magnetic resonance imaging (MRI) within 10 min and ultimately leads to further compression of the injured spinal cord.^10,11^ The swollen spinal cord becomes compressed circumferentially and longitudinally against the dura mater and rigid bone at the injury epicenter and beyond, resulting in the displacement of cerebrospinal fluid and the effacement of the subarachnoid space across multiple vertebral segments.^7,11–13^

Compression of the spinal cord against an unyielding spinal canal results in increased intraspinal pressure and reduced perfusion pressure, further jeopardizing blood flow to the spinal cord.^14–16^ In motor complete TSCI patients, swelling of the spinal cord spreads rostrally and caudally from the injury epicenter in fusiform fashion,^8^ at a rate of ∼900 μm/h.^13,17^ Frequently, by the time the victim is transferred to the trauma center, intramedullary lesion length (IMLL), can measure between 40 and 100 mm in length, far beyond the cross-sectional injury epicenter.^18–20^ Pre-clinical studies indicate that the longer the spinal cord is compressed, the more severe is the parenchymal damage and loss of function.^21–24^ Together, these findings have encouraged many trauma spine surgeons to recommend early operative intervention as a neuroprotective measure in order to promote neurological improvement.^25–30^

Ongoing surgical studies on the relationship among imaging biomarkers, surgical strategy, and long-term clinical outcome are shaping the standard of care for these patients.^18,28,31–36^ Over the past 30 years, overarching evidence has supported spinal cord decompression following trauma, but the question of surgical timing has yet to be established within this evolving paradigm.^10,12,32,35,37–41^ In addition, although multiple reports have indicated that early anatomical alignment of the spinal column and decompression of the spinal cord followed by internal fixation is neuroprotective, in these studies, decompression of the spinal cord generally has not been verified by post-operative imaging.

Recent efforts have begun to establish the significance of the extent of spinal cord decompression in neurological outcome.^16,35,42,43^ Data reported by Aarabi and coworkers indicated that standard surgical management of cervical SCI achieves decompression of the entire swollen segment of the spinal cord in only 66% of subjects.^35^ Additional studies incorporating post-operative MRI indicate that up to 25% of patients may need expansive duraplasty for adequate spinal cord decompression, along with reduction of intraspinal pressure, in order to improve functional outcome.^14,15,42,44,45^ As a result, studies on the effect of timing of surgical intervention have likely been confounded by incomplete spinal cord decompression, and, therefore, the effect of timing on outcome remains uncertain.^28,30,46–48^

The null hypothesis was that in patients with subaxial cervical TSCI and post-operative MRI confirmation of decompression, the timing of surgery has no effect on long-term improvement in American Spinal Injury Association (ASIA) Impairment Scale (AIS) grade conversion.

## Methods

### Design

This study was a retrospective analysis of prospectively collected data.

### Cohort

Over a 13-year period, from January 1, 2005 to December 31, 2017, 950 MRI-proven isolated cervical TSCI patients were admitted to this level I trauma center for management. From this cohort, we screened and selected 72 patients who were eligible for this investigation. The *inclusion criteria* were being ≥16 years of age; Glasgow Coma Scale (GCS) score ≥14; no concurrent life-threatening injury or disease; imaging studies compatible with subaxial cervical spine fracture dislocations; available good quality pre- and post-operative computed tomography (CT) and MRI studies indicating complete spinal cord decompression following surgery;^35^ and follow-up of at least 6 months after trauma and surgical management. The *exclusion criteria* were being obtunded, stuporous, and non-testable; having penetrating subaxial TSCI; having upper cervical SCI; a post-operative MRI indicating inadequate spinal cord decompression; non-operative management; having had a cervical CT myelogram and not an MRI as the primary imaging study; dying or being lost to follow-up; or having poor-quality imaging studies. This study was performed after approval from the institutional review board (IRB) of the Human Research Protection Office (HRPO).

### Resuscitation, survey, and neurological examination

Patients were transferred to the trauma resuscitation unit (TRU) by emergency medical technicians (EMTs).^49^ We received intubated and non-intubated patients supine on a backboard with the head and neck secured with a hard collar and chin strap. Median scene or transfer time after the accident was 1 h (mean, 2.3; standard deviation [SD], 3; range, 0.3–15 h). In the TRU, primary and secondary examinations were performed by one of three teams of trauma surgeons who received the patients. Once the patients were medically stable, members of the neurosurgical team (senior resident or nurse practitioners) first examined and then presented them to the attending neurosurgeon. Admission ASIA motor score and AIS grade were determined according to the International Standards for Neurological Classification of Spinal Cord Injury (ISNCSCI).^50^ ASIA motor scores and AIS grades, which were used for statistical calculation were the ones with no effect from sedatives, analgesics, or mental confusion. In the majority of our patients, the TRU examination was definitive; however, in a minority of patients, the neurological examination within the first 72 h following trauma was used once the sedatives, analgesics and anesthetics were cleared from the patient's body.

### Imaging studies

Eligible patients had imaging studies performed when they were medically stable. Cervical spine CT was performed within a median of 2 h (mean, 3.2; SD, 3.1; range, 3.5–15.8 h) and MRI was performed within a median of 5.8 h (mean, 7.2; SD, 5.1; range, 2.4–39.5 h) from the time of the accident. The median time between the accident and MRI was 5 h for the ultra-early patients (mean 5.9 h, range 3–12 h), 6 h for the early patients (mean 6.8 h, range 2.9–14 h), and 6 h for the late patients (mean 10.4 h, range 3.5–39.5 h). Fracture morphology was based on the Harris and coworkers,^51^ Allen and coworkers,^52^ and AO Spine^53^ classification systems. Admission T2-weighted and short T1 inversion recovery (STIR) sequences were used to measure the IMLL and the extent of spinal cord compression/decompression before and after surgery.^18,35^ An attending trauma neuroradiologist and the principal investigator independently measured the IMLL, and the mean value was taken for statistical analysis.^18^

### Medical management

From 2005 to 2009, 22 of the study patients were administered methylprednisolone following SCI: 30 mg/kg within the first hour and 5.4 mg/kg/h for the next 23 h.^54^ In 2010, this trauma center stopped the use of steroids for SCI. Patients' mean arterial blood pressure was maintained between 85 and 90 mm Hg for 7 days following trauma.^54,55^

### Traction and surgical intervention

When indicated, we applied traction for the reduction of cervical spine deformities immediately following CT and/or MRI studies.^35,56^ We set up traction in the TRU, on a Stryker Wedge Turning Frame (Stryker Global Headquarters, Kalamazoo, Michigan) using real-time fluoroscopy. We applied incremental weights of 2–4 kgs per skeletal segment within a maximum of 30–45 min. If this was not successful, we reduced the deformity during surgery. Twenty patients had traction for unilateral (*n* = 3) or bilateral (*n* = 14) jumped facets or for compression fracture with >3 mm translation (*n* = 3). We did not apply skeletal traction in 52 patients for several reasons: no evidence of fracture dislocation (*n* = 17); flexion or extension injury without translation (*n* = 20); compression fracture with <3 mm translation (*n* = 12); or unilateral locked facets (*n* = 3).

### Surgical management

Patients were decompressed and internally fixed within a median of 12 h (mean, 18.8; SD, 19.4; range, 4.5–138.5 h). Thirty-two (44.5%) patients were operated on in <12 h, 25 (34.7%) were operated on within 12–24 h, and 15 (20.8%) were operated on >24–138.5 h after the trauma. Nine neurosurgeons including four spine fellowship-trained neurosurgeons performed the surgeries. Surgeries included anterior cervical discectomy and fusion (ACDF) in 10, ACDF+laminectomy in 25; anterior cervical corpectomy and fusion (ACCF) in 9; ACCF+laminectomy in 11; and only laminectomy in 17.^35,46,57^ Post-operative CT and MRI were performed to confirm appropriate technique and to verify spinal cord decompression. Post-operative MRI studies were performed within a median of 34 h (mean, 45.1; SD, 30.8; range, 13.5–148.5 h). Post-operative MRI in the ultra-early group was performed a median of 31.5 h following trauma (mean 34.2, range 13.5–95.5 h). In the early group, the median time following trauma was 29.8 h (mean 42.4 and range 18.8–139.5 h). In the late group, the median time following trauma to MRI was 56.9 h (mean 72.9 and range 39.8–148.5 h). Two spine fellowship-trained neurosurgeons, one fellowship-trained neurotrauma neurosurgeon, a trauma neuroradiologist, and the principal investigator independently verified complete spinal cord decompression on postoperative MRI studies.^35^ Spinal cord decompression was defined as presence of cerebrospinal fluid in the subarachnoid space around the spinal cord circumferentially in all patients.

### Post-operative ICU care and follow-up

The post-operative course in the ICU included deep vein thrombosis (DVT) prophylaxis by enoxaparin (Lovenox^®^, Sanofi, USA), 30 mg twice daily starting within 24–48 h of admission, and screening by duplex ultrasound for venous thromboembolism (VTE). When needed, patients had early tracheostomy for ventilator support and percutaneous gastroenterostomy for nutrition. When fully weaned from ventilator support, the patients were transferred to rehabilitation centers. While in the ICU, daily neurological examination including digital rectal examination was performed to determine ASIA motor score and evidence for AIS grade conversion. Following discharge, the patients returned at 6 weeks, 3 months, 6 months, and 12 months (or longer) for follow-up neurological examinations. Certified neurologists, rehabilitation specialists, the principal investigator, senior residents, and nurse practitioners performed neurological examinations to document any change in ASIA motor score and AIS grade conversion.^18,35^

## Results

### Initial patient characteristics

Age, gender, injury mechanism, IMLL, ASIA motor score, and surgical technique did not differ significantly among the three groups of patients: those with ultra-early (< 12 h), early (12–24 h), or late (> 24–138.5 h) decompression (Table 1). Compared with AIS grade C patients, AIS grade A and B patients were operated on significantly earlier after admission (*p* = 0.009). Similarly, patients with burst/compression fractures and those with facet dislocations (types A3/A4 and C of the Vaccaro and coworkers AO Spine classification^53^) were surgically managed earlier than patients with no evidence of fractures and horizontal translation (AO Spine Class types A0, B2, B3) on CT and MRI (*p* = 0.01)

**Table 1.** Baseline Characteristics of the Present Cohort

Category	<12 h post-trauma	12-24 h post-trauma	24-138.5 h post-trauma	Total	*p*
Accident (%)					0.59
Fall	14 (38.9)	14 (38.9)	8 (22.2)	36 (50)	
MVC	12 (60)	5 (25)	3 (15)	20 (27.8)	
Other	6 (37.5)	6 (37.5)	4 (25)	16 (2.2)	
Total	32 (44.4)	25 (34.7)	15 (20.8)	72 (100)	
Gender (%)					0.89
Male	26 (43.3)	21 (35)	13 (21.7)	60 (83.3)	
Female	6 (50)	4 (33.3)	2 (16.7)	12 (16.7)	
Total	32 (44.5)	25 (34.7)	15 (20.8)	72 (100)	
Age (years): Mean (SD)	41.8 (18.4)	49.4 (18.3)	49.3 (13.2)	46.0 (17.6)	0.19
AIS grade (%)					0.009
AIS A	13 (48.2)	11 (40.7)	3 (11.1)	27 (37.5)	
AIS B	14 (60.9)	7 (30.4)	2 (8.7)	23 (31.9)	
AIS C	5 (22.7)	7 (31.8)	10 (45.5)	22 (30.6)	
ASIA motor score: Mean (SD)	18.6 (14.4)	22.0 (15.2)	24.5 (14.2)	21.1 (14.6)	0.40
Morphology (%)					0.01
A0	5 (20)	12 (48)	8 (32)	25 (34.7)	
A3/A4/C	24 (61.5)	11 (28.2)	4 (10.3)	39 (54.2)	
B2/B3	3 (37.5)	2 (25)	3 (37.5)	8 (11.1)	
Total	32 (44.5)	25 (34.7)	15 (20.8)	72 (100)	
IMLL (mm): Mean (SD)	43.3 (19.5)	37.5 (17.9)	30.6 (13.9)	38.6 (18.4)	0.07
Surgical technique (%)					0.32
ACDF	5 (50)	2 (20)	3 (30)	10 (13.9)	
ACDF+Laminectomy	10 (40)	11 (44)	4 (16)	25 (34.7)	
ACCF	7 (77.8)	2 (22.2)	0 (0)	9 (12.5)	
ACCF+Laminectomy	5 (45.4)	4 (36.4)	2 (18.2)	11 (15.3)	
Laminectomy	5 (29.4)	6 (35.3)	6 (35.3)	17 (23.6)	
Total	32 (44.4)	25 (34.7)	15 (20.8)	72 (100)	

MVC, motor vehicle crash; SD, standard deviation; AIS, American Spinal Injury Association (ASIA) Impairment Scale; IMLL, intramedullary lesion length; ACDF, anterior cervical discectomy and fusion; ACCF, anterior cervical corpectomy and fusion.

### AIS grade conversion

**FIG. 1.**
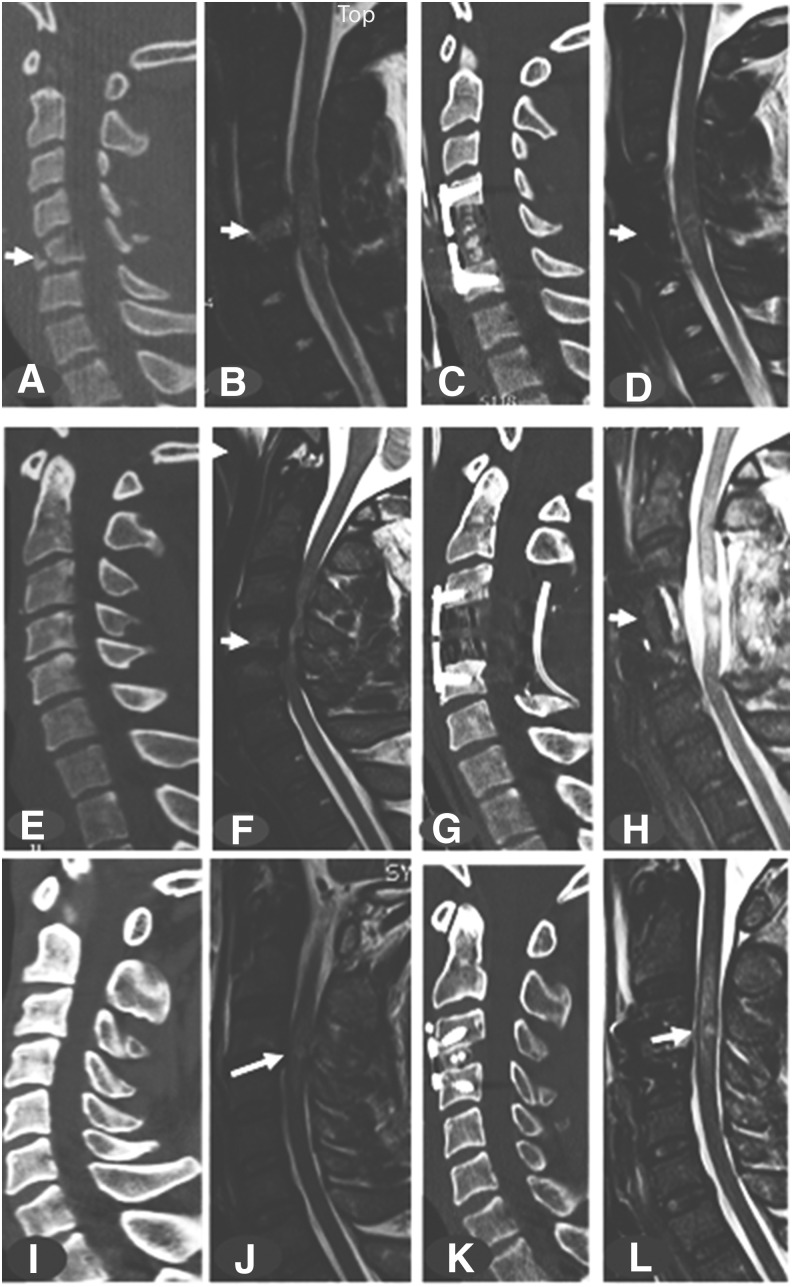
**(A–D)** Midsagittal computed tomography (CT) and magnetic resonance imaging (MRI) of a 19-year-old man involved in an automobile accident who was admitted 30 min later to the trauma resuscitation unit (TRU) with a C5 compression tear-drop fracture (arrow); American Spinal Injury Association (ASIA) motor score of 21, and ASIA Impairment Scale (AIS) grade B; intramedullary lesion length (IMLL) at admission was 27.8 mm. A C5 corpectomy was performed 6 h after the accident, which completely decompressed the spinal cord. MRI 34.5 h after surgery indicated an IMLL of 34.4 mm. One year following the accident, his ASIA motor score was 64 and he was AIS grade D. **(E–H)** Midsagittal CT and MRI of a 42-year-old man who had a mechanical fall and was admitted 30 min later to the TRU with from spinal stenosis and possible extension injury (arrow); ASIA motor score was 8 and AIS grade was A; IMLL at admission was 42.2 mm. He underwent C4 corpectomy and C3–C5 laminectomy with fusion 13 h after the accident, with complete spinal cord decompression. MRI 23 h after surgery indicated an IMLL of 30.9 mm. Six months following the accident, his ASIA motor score remained 8 and he was AIS grade A. **(I–L)** Midsagittal CT and MRI of a 53-year-old man who had a mechanical fall and was admitted 10.5 h later to the TRU with a C3/4 extension injury (arrow); ASIA motor score was 33 and AIS grade was C; IMLL at admission was 20.3 mm. He underwent discectomy and fusion at C3/4, 36 h after the accident, with complete spinal cord decompression. MRI 49.8 h after surgery indicated an IMLL of 49.6 mm. Fifty-seven months following the accident, his ASIA motor score was 91 and he was AIS grade D.

During a period of at least 6 months of follow-up, three patients had regression of their AIS grade compared with at admission. In the ultra-early group, two patients in the AIS grade B group converted to AIS grade A, and in the early group, one patient in the AIS grade C group regressed to AIS grade B (Tables 2–6). The effect of nine independent variables on AIS grade conversion when the spinal cord was completely decompressed on post-operative MRI was analyzed. From the demographic variables, neither age (*p* = 0.07) nor gender (*p* = 0.18) had any influence on AIS grade conversion. The mechanism of injury also was without effect on neurological outcome (*p* = 0.638). Patients with higher ASIA motor score at admission had a better rate of conversion at 6 months (*p* < 0.0004), but the rate of conversion was not influenced by admission AIS grade (*p* = 0.058). One reason for lack of congruity between ASIA motor score and AIS grade could be the fact that AIS grade is a composite of ASIA motor score, sensation, and sacral sparing, which could have unknown confounding statistical effects on each other. Regarding the CT scan of the cervical spine, regardless of whether there were no fracture/dislocations (A0) or fracture/dislocation along one or multiple axes (AO Spine classification morphology types A3/A4, B2/B3, C), morphology type did not affect grade conversion (*p* = 0.11).^53^ Five different surgical techniques were used for complete decompression of the spinal cord.^35^ The type of surgical technique did not affect neurological outcome (Fig. 1). Contrary to much of the literature (see Table 7), surgical decompression in any of the three time periods had no effect on improvements in AIS grade conversion (*p* = 0.424).^28,47,58,59^ Intramedullary lesion length (IMLL) on T2W or STIR images, as a surrogate end-point biomarker, had the most powerful effect on AIS grade conversion (p = 0.001).^18,35,60–62^ Compared with patients who had no improvement in AIS grade, patients with grade conversion had significantly shorter IMLL on pre-operative T2W or STIR MRI images. Regression analysis of variables with no effect, marginal effect, or significant effect on AIS grade conversion indicated that IMLL had the most powerful influence on improved grade conversion at 6 months following trauma (Table 6).

**Table 2.** AIS Grade Conversion in 32 Patients with Ultra-Early (< 12 h) Decompression

						

^*^ AIS grade regression.

AIS, American Spinal Injury Association (ASIA) Impairment Scale.

**Table 3:** AIS Grade Conversion in 25 Patients with Early (12-24 h) Decompression

						

^*^ AIS grade regression.

AIS, American Spinal Injury Association (ASIA) Impairment Scale.

**Table 4.** AIS Grade Conversion in 15 Patients with Late (>24-138.5 h) Decompression

	AIS grade at follow-up					
Admission AIS grade	A	B	C	D	E	Total admission
						AIS grade
A	1	1	0	1	0	3
B	0	1	0	1	0	2
C	0	0	1	9	0	10

AIS, American Spinal Injury Association (ASIA) Impairment Scale.

**Table 5.** AIS Grade Conversion in Various Categories of the Cohort

Category	AIS not converted	AIS converted	Total	*p* value
Accident (%)				0.638
Fall	12 (33.3)	24 (66.7)	36 (50)	
MVC	8 (40)	12 (60)	20 (27.8)	
Other	4 (25)	12 (75)	16 (22.2)	
Total	24 (33.3)	48 (66.7)	72 (100)	
Gender (%)				0.18
Male	22 (36.7)	38 (63.3)	60 (83.3)	
Female	2 (16.7)	10 (83.3)	12 (16.7)	
Age (SD)	40.8 (16.2)	48.6 (17.9)	46.0 (17.6)	0.07
Admission AIS grade (%)				0.058
A	12 (44.4)	15 (55.6)	27 (37.5)	
B	9 (39.1)	14 (60.9)	23 (31.9)	
C	3 (13.6)	19 (86.4)	22 (30.6)	
Total	24 (33.3)	48 (66.7)	72 (100)	
Admission ASIA motor score (SD)	12.7 (12.5)	25.2 (13.9)	21.0 (14.6)	0.0004
Morphology (%)				0.11
A0 (No evidence of fracture dislocation)	6 (24)	19 (76)	25 (34.7)	
B2 or B3 (Flexion or extension injury)	1 (12.5)	7 (87.5)	8 (11.1)	
A3/4+C (Significant translation in X/Y/Z axes)	17 (43.6)	22 (56.4)	39 (54.2)	
Intramedullary lesion length (IMLL) (mm, SD)				
Admission grade A	59.3 (20.7)	35.6 (9.6)	46.1 (19.4)	0.002
Admission grade B	54.2 (19.1)	31.9 (11.9)	40.6 (18.4)	0.003
Admission grade C	23.9 (17.1)	27.9 (9.9)	27.4 (10.6)	0.472
Total	53.0 (22.1)	31.5 (10.7)	38.6 (18.4)	0.00001
Surgical intervention (%)				NS
ACDF	3 (30)	7 (70)	10 (13.9)	
ACDF+Laminectomy	5 (20)	20 (80)	25 (34.7)	
ACCF	1 (11.1)	8 (88.9)	9 (12.5)	
ACCF+Laminectomy	9(81.8)	2 (18.2)	11 (15.3)	
Laminectomy	6 (35.3)	11 (64.7)	17 (23.6)	
Timing of surgery (%)				0.424
<12 h after trauma	11 (34.4)	21 (65.6)	32 (44.5)	
12-24 h after trauma	10 (40)	15 (60)	25 (34.7)	
>24 h after trauma	3 (20)	12 (80)	15 (20.8)	
Total	24 (33.3)	48 (66.7)	72 (100)	

AIS, American Spinal Injury Association (ASIA) Impairment Scale; MVC, motor vehicle crash; SD, standard deviation; ACDF, anterior cervical discectomy and fusion; ACCF, anterior cervical corpectomy and fusion.

**Table 6.** Multivariate Regression Analysis Comparing the Therapeutic Efficacy of Timing of Surgery Versus Intramedullary Lesion Length (IMLL)

Outcome	Odds ratio	95% confidence interval	*p* value
<12 h trauma-surgery	Referent	-	-
12-24 h trauma-surgery	0.455	0.118-1.752	0.25
>24 h trauma-surgery	0.832	0.141-4.88	0.83
IMLL (mm)	0.908	0.862-0.957	0.001

**Table 7.** Investigations Evaluating the Timing of Decompression on the Therapeutic Effectiveness and Neurological Outcome in Cervical Traumatic Spinal Cord Injury

Investigator**Year Journal	Design	Cohort	AIS grade	Preop MRI	Timing (hours)	Postop MRI	IMLL	Extent of DEC.	F/UM	TE/AIS grade conversion
Vaccaro et al. Spine, 1997	PR	62	A-D	Yes	≤72 and >120	No	No	No	11.5	No effect
Guest et al. J. Neurosurg., 2002	RO	50	C-D	Yes	≤24 and >24	No	No	No	36	Not mentioned
Papadopoulos et al. J. Trauma 2002	PO	91	A-D	Yes	<12 and >12	No	No	No	33	Early superior
Sapkas and Papadakis J. Orthop. Surg. 2007	RO	67	A-E	Yes	≤72 and >72	No	No	No	48	No effect
Lenehan et al. Spine 2010	PO	73	C-D	Yes	≤24 and >24	No	No	No	12	No effect
Wilson et al. Spinal Cord 2012	PO	55	A-D	Yes	<24 and ≥24	No	No	No	3	Early superior
Fehlings et al. PLoS One 2012	PO	313	A-D	Yes	<24 and ≥24	No	No	No	6	Early superior
Jug et al. J. Neurotrauma 2015	PO	42	A-C	Yes	≤8 and 8-24	No	No	No	6	Early superior
Dvorak et al. J. Neurotrauma 2015	PO	470	A-D	Yes?	≤24 and >24	No	No	No	3-6	NM
Grassner et al. J. Neurotrauma 2016	RO	70	A-D	Yes	≤8 and 8-90	No	No	No	10	Early superior
Bourassa-Moreau et al. J. Neurotrauma 2016	PO	20	A	Yes	<24-≥24	No	No	No	5	Early superior
Mattiassich et al. J. Neurotrauma 2017	RO	49	A-D	Yes	<5 h and ≥5-24	No	No	No	6	Late superior
Burke et al. Neurosurgery 2018	RO	48	A-D	Yes	≤12,12-24,>24	No	No	No	ACD	Early superior
Kim et al. World Neurosurg. 2018	RO	46	A-D	Yes	≤48 and >48	No	No	No	6	No effect
Sewell et al. World Neurosurg. 2018	RO	95	A-D	Yes	≤24 and >24	No	No	No	6	No effect
Current Study 2019	RO	73	A-C	Yes	≤12,12-24,24-138.5	Yes	Yes	Yes	6	No effect

AIS, American Spinal Injury Association (ASIA) Impairment Scale; MRI, magnetic resonance imaging; IMLL, intramedullary lesion length; ACD, acute care discharge; DEC, decompression; F/U, follow-up; M, months; PO, prospective observational; PR, prospective randomized; RO, retrospective observational; TE, treatment effect.

## Discussion

The principal finding of the present study is that, in a cohort of patients with cervical TSCI with pre-operative MRI evidence of spinal cord compression and post-operative MRI evidence of complete decompression, it was the IMLL, not the timing of surgery, that best predicted improved AIS grade conversion.

A meta-analysis of pre-clinical studies^63,64^ on experimental TSCI concluded that neuroprotection may be obtained when decompression is performed early following injury. However, in the studies subjected to meta-analysis, the degree of compression was difficult to compare directly because of important methodological differences, with some studies introducing spacers to narrow the canal diameter, others compressing the cord with aneurysm clips or weights exerting different forces, and others compressing the cord with devices exerting known pressures. In a rodent pre-clinical study by Batchelor and coworkers, a sustained compression force of ∼35 mm Hg quickly resulted in paraplegia.^65^ In a meta-analysis by the same investigators, early decompression improved behavioral outcome in rodents and non-human primates by up to 35%.^64^ Although supportive of early decompression, the pre-clinical studies included in Batchelor's meta-analysis were heterogeneous in design, methodology, and timing of decompression, and these studies suffered from low internal validity and reporting bias. Translating data from pre-clinical models to humans has repeatedly proven to be very difficult.^63,64^

The therapeutic effectiveness of timing (early vs. late) of surgical decompression and its relationship with neurological outcome following cervical TSCI is at equipoise (Table 7).^28,30,59,66,67^ Heterogeneity in design, methodology, surgical technique, neurological assessment, timing of decompression, number of patients enrolled, length of follow-up, and outcome assessment tools used makes interpretation of the results difficult and the therapeutic effectiveness of timing of decompression uncertain. Notably, most of the studies published during the past 20 years have confirmed pre-operative spinal cord compression by CT or MRI, but seldom has there been a precise, anatomic definition of the term *decompression*, the purported goal of surgery. In many studies, the surgical technique, including the number of discectomies, corpectomies, or laminectomies in isolation or in combination required to decompress the swollen spinal cord was not well documented.^28,30,46,47,68^ Almost universally, decompressive surgery was not followed by post-operative MRI to verify complete decompression of a swollen spinal cord.^28,29,47,48,58,59,66–73^ By contrast, Papadopoulos and coworkers,^47^ and Sapkas and Papadakis,^67^ used MRI following closed traction reduction to assess for any residual compression following anatomical realignment and to decide on the need for further spinal cord decompression. In the multi-center prospective observational study by Fehlings and coworkers,^28^ postoperative MRI was performed only in patients who had neuroworsening following surgical decompression. These studies uniformly used CT scan evidence of injury morphology as a guide for surgical intervention, not the IMLL or the extent of swelling across several skeletal segments present on preoperative MRI. In a recent clinical study, standard surgical approaches as recommended by Dvorak and coworkers^57^ were successful in decompressing the swollen spinal cord in only 66% of AIS grade A and B patients.^35^ Another study in AIS grade A, B, and C patients indicated that complete decompression resulted in AIS grade conversion in 58.9% of patients, whereas inadequate decompression resulted in improved AIS grade conversion in only 18.6% of patients.^18^ Adequate laminectomy is the procedure that is the *sine qua non* for assured spinal cord decompression.^35^ In the latter series, almost 74% of patients had to have multi-level laminectomies for adequate decompression of the swollen spinal cord. By contrast, the rate of laminectomy in the randomized study of Vaccaro and coworkers^30^ was 9.6%, and in the prospective observational study of Jug and coworkers,^29^ it was 7%. Therefore, the surgical methodology in these two series could have confounded the interpretation of the effect of timing of decompression on neurological outcome. In published articles discussing the timing of decompression and neurological outcome, MRI-documented IMLL, an important predictor of neurological outcome, was not used to guide the extent of surgery required for adequate spinal cord decompression.^18,20,61,62,74–78^

In addition to imaging biomarkers such as structural MRI, which convey a visual indication of compression, studies have revealed the importance of juxtaluminal pressure measurements inside the parenchyma of the spinal cord.^42,43,79^ The presence of a swollen spinal cord against nonyielding dura may indicate a requirement for expansive duraplasty.^43^ In the current study, 100% of the patients had an open subarachnoid space (as a prerequisite inclusion criterion); therefore, the need for a juxtaluminal pressure monitor is less clear. However in a previous report from the University of Maryland, only 7 of 63 patients (11%) with inadequate bony decompression also exhibited effaced subarachnoid space, therefore being candidates for intraspinal pressure monitoring and expansive duraplasty.^35^ Consideration of intraspinal pressure and spinal cord perfusion pressure and pressure reactivity index may help improve neurological outcomes following inadequate bony decompression performed within the appropriate time window following trauma.^22,45,80,81^

Apart from structural MRI, which is mandatory for the diagnosis, pre-operative planning, estimation of the degree of spinal cord compression, IMLL, and the extent of spinal cord decompression, quantitative MRI (QMRI) biomarkers including magnetization transfer, relaxation mapping, and diffusion imaging have helped us better understand secondary changes following TSCI at a microstructural level across the entire neuraxis.^36, 82–87^ QMRI depicts the extent of iron deposition in the spinal cord and brain and the degree of demyelination and degeneration, and it can predict neurological outcome and the degree of neural plasticity.^36,83^ These studies may be useful as surrogate end-points in trials on the timing of decompression and AIS grade conversion following TSCI.^88–90^

The present study underscores the emerging importance of injury severity parameters such as IMLL and Brain and Spinal Injury Center (BASIC) score,^77^ as well as the extent of decompression, in studies involving the timing of decompression. If future multi-center and prospective studies take into account IMLL and completeness of decompression, the actual effect of the timing of decompression in TSCI will likely be clarified.

Limitations of this study include the following.

1. The majority of the motor complete TSCI patients were in the ultra-early and early decompression categories, whereas the reverse was true for motor incomplete patients (AIS grade C) who were in the late decompression category.
2. In a minority of our patients, ISNCSCI examination, which was used for statistical analysis, was completed within 72 h of trauma when the effects of analgesics, sedatives, and anesthesia were absent and there was no mental confusion.

## Conclusion

Pre-clinical studies, biological rationale, and recent clinical investigations favor early spinal cord decompression to enhance motor outcome following TSCI. Many of the clinical trials to date have been heterogeneous, biased, and with low internal validity. These studies have been based on standard surgical procedures that rely on pre-operative CT and MRI, with no independent verification of the completeness of spinal cord decompression on post-operative MRI. In our study, when the spinal cord was shown to be decompressed on post-operative MRI, the timing of decompression did not influence AIS grade conversion. Here, we identified intramedullary lesion length as the main predictor of neurological outcome.
